# Cultivation of humanistic values in medical education through anatomy pedagogy and gratitude ceremony for body donors

**DOI:** 10.1186/s12909-020-02292-1

**Published:** 2020-11-17

**Authors:** Kaihua Guo, Tao Luo, Li-Hua Zhou, Dazheng Xu, Guangming Zhong, Huaqiao Wang, Jie Xu, Guoliang Chu

**Affiliations:** 1 Department of Anatomy, Zhongshan School of Medicine, Sun Yat-Sen University, Guangzhou, 510080 People’s Republic of China; 2 Department of Anatomy, Sun Yat-sen University School of Medicine, Sun Yat-Sen University, Guangzhou, 510089 People’s Republic of China

**Keywords:** Humanistic qualities, Medical education, Human anatomy, Ethics, Silent mentor

## Abstract

**Background:**

One of the most important objectives of modern medical education is to empower medical students to become humanistic clinicians. Human anatomy plays a crucial role in this mission by using cadavers to cause reflections on death, dying, illness, and the role of medical practitioners in humanistic care. The objective of this study was to introduce, describe, and evaluate the impact of a ceremony in honor of the body donors on ethical and humanistic attitudes of medical students.

**Methods:**

We used a phenomenological research approach to explore and understand the lived experiences of the anatomy teachers as they teach anatomy in the context of humanism and ethics. A separate survey of third-year medical students was carried out to understand their perceptions of changes in themselves, respect for donors and donor families, and their relationship with patients. Data were collected in two phases: a desktop review of teaching materials followed by in-depth interviews of the main anatomy teachers followed by a self-administered, 5-item Likert scaled questionnaire given to students.

**Results:**

In the present article, we describe the rituals conducted in honor of body donors at our School of Medicine. We also describe the lived experiences of anatomy teachers as they work on improving humanistic education quality through the introduction of the concept of “silent mentor” which refers to a cadaver that quietly allows medical students to learn from it. In turn, a ceremony in honor of body donors who have altruistically donated their bodies so that learning anatomy through dissection would be possible is also introduced. A survey of the impact of the ceremony in honor of body donors on medical students revealed positive responses in terms of promoting studying anatomy (3.96 Vs 3.95) as well as reflections on own death (4.44 Vs 4.35), the life of body donors (4.07 Vs 4.04), and how to humanely view future patients and their significant others (4.32 Vs 4.24) relative to those that did not attend the ceremony (5-item Likert scale). The majority of the students that attended the ceremony also indicated that it had a positive impact on their future doctor-patient relationship, thinking about the possibility of donating their body for teaching as well as about medical ethics. Most of them also think that attending the ceremony helped reduce their anxiety, fear, and disgust of seeing corpses or dissecting and 90% insisted that memorial ceremonies should continue being conducted at Zhongshan Medical School.

**Conclusion:**

The combination of the anatomy component of the basic medical curriculum and gratitude ceremonies as well as activities to promote body bequeathal programs might help to accomplish the goal of cultivating high-quality medical students and professionals for the future. The long-term benefits would be a medical graduate who exudes empathy, relates well with patients and their significant others, leading to a productive doctor-patient relationship.

**Supplementary Information:**

The online version contains supplementary material available at 10.1186/s12909-020-02292-1.

## Background

The modern history of medical humanities is rooted in crises such as revelations of routine research abuses, rising cost, and exclusiveness that rocked medicine in the twentieth century. During this time some American teachers and preachers were concerned about “depersonalization,” “the centrality of molecular biology,” and “the teaching of mechanistic medicine” which they attributed to disrupting relationships between doctors, patients, and society [1]. Amid the challenges to clearly defining the role and definition of humanism as applied to the practice of medicine, the vision to reconnect humanistic and scientific ways of “knowing” as well as “being” gained traction all over the world. In 2008, the Chinese Ministries of Education and that of Public Health issued a joint “Undergraduate Medical Education Standard-clinical medicine specialty” (proposed regulations) policy document which contained a set of standards structured according to three areas namely: ethics and occupational quality, knowledge objective, and technical objective. All these standards emphasized the importance of ethics and humanistic education. The humanistic education standards were set to encompass a society filled with appreciation, gratitude, ultimate concern, cherishing life, revering life, fairness, justice, the meticulous attitude among other attributes. As anatomy teachers, we have sought to infuse these attributes into the anatomy course involving human cadaver dissection.

The contemporary medical care system in China is in a stage of continuous transformation, especially on the humanistic values and ethics front. China’s large patient population far exceeds the limited resources and available medical services. There are frequent media reports on medical workers who “treat life with indifference, but organs with a difference”. This mechanistic phenomenon exposes the deficiency in medical ethics and a lack of a conducive care atmosphere. Furthermore, traditional medical education models over-emphasize the acquisition of professional knowledge and skills whilst neglecting the cultivation of humanistic values and medical ethics [2]. It is against this background that we sought to introduce the humanistic and medical ethics issues early on in the medical curriculum. In anatomy, cadaver dissection has largely been connected with the concept of “detached concern” in which medical students were encouraged to emotionally disconnect themselves with the cadavers and simply focus on mastering anatomy content [3]. These traditional models certainly result in poor insight and social adaptation among medical graduates. As a consequence, there is a high likelihood of the entire breadth of the medical service failing to satisfy society’s health needs.

In China, the nature and characteristics of the tense relationship between patients and doctors have been widely discussed in the literature [4–6]. In some cases, the tense relationship has resulted in violence towards doctors [7]. This necessitates the inculcation of humanistic values among graduating doctors. In today’s humanism, several disparate disciplines, including anatomy, are held together by their importance to medicine. Anatomy education presents medical students with cadavers as their “first patients”. This opportunity can come with greater emphasis on the cultivation of humane attributes such as compassion, care, and empathy among prospective medical doctors, so as to counteract the concept of “detached concern” [8–10]. The concept of the “first patient” blends well with that of “silent mentors” in which cadavers (donated bodies) act as teachers (mentors) that “quietly” afford medical students a fundamental learning aid through dissections [11]. As a result, medical students begin to revere their cadavers by following dissection room rules and handling them with care, thereby getting primed for humanistic value inculcation. Therefore, in this study, we will advance the view that teaching humanistic values and ethics can be integrated well in gross anatomy pedagogy at entry-level learning through the experiences of anatomy teachers who oversee extensive medical student- -cadaver contact. Chinese medical colleges have faced cadaver shortages due to a conservative culture, only recently introduced body donation programs, and also been called upon to improve on these body bequeathal programs [12, 13]. Since the quantitative analysis of the impact of ceremonies on medical students remains scarce [11, 14], the other aim of this study is to assess the impact of a ceremony held in honor of the body donors on ethical and humanistic attitudes of medical students.

## Methods

The Research Ethics Committee of the Sun Yat-sen University approved this study before execution. Our School of Medicine is over 120-years old and has aimed to produce graduates who exhibit a very high level of responsibility to the nation and also embrace international perspectives in their conduct. This two-part study was set at the Zhongshan School of Medicine at Sun Yat-sen University to understand the involvement of anatomy teachers in humanistic education as well as evaluation of feedback of such a program.

The first part of this study was grounded in phenomenology which focuses on describing particular phenomena, or the appearance of things, as lived experiences [15]. The phenomenological research approach was used to explore and understand the lived experiences of the anatomy teachers as they conduct the ceremony to honor body donors and also teach the subject while relating it to humanistic medical practice. The phenomenological approach involved in-depth interviews of the main professors involved in anatomy teaching. The interviews enabled obtaining in-depth reflections on how the ceremony to honor body donors, as well as anatomy teaching methods and materials used, are of relevance to ethical clinical practice. The non-probability purposive sampling design was used for the study in which the main professors from the said university were interviewed. We used a critical narrative analysis in which the findings are discussed in relation to pertinent literature around anatomy pedagogy and medical ethics.

### A memorial ceremony for “silent mentors”

**Fig. 1:**
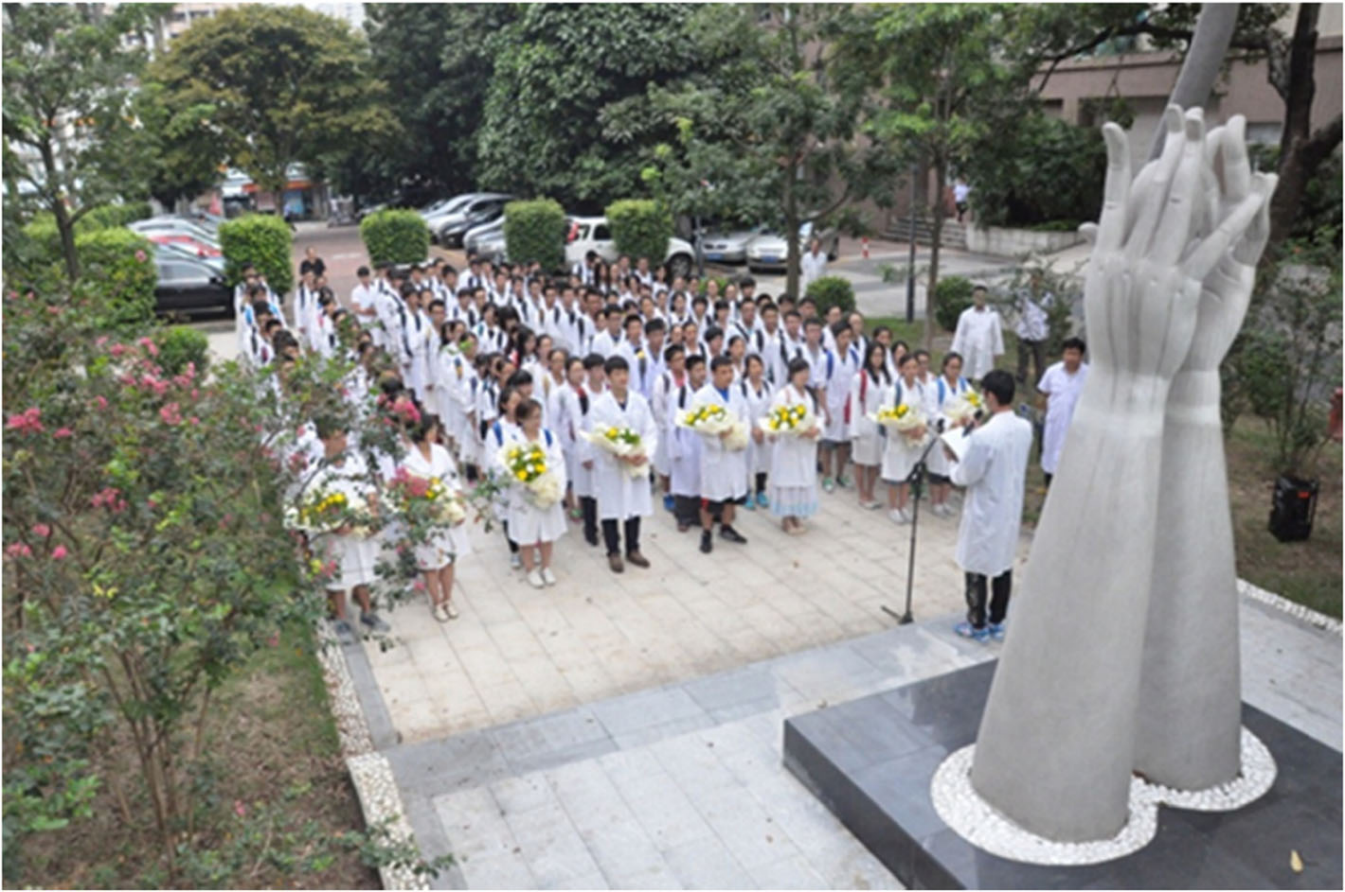
A salute to “silent mentors” is conducted every year at the specially built Square of Perfect Goodness at our School of Medicine

“Silent mentors” is often used to refer to the donated bodies used for dissections during anatomy learning. A salute to “silent mentors” is conducted yearly at the beginning of the Human Anatomy course at our School of Medicine. This solemn ceremony is held at the specially built area called the “Square of Perfect Goodness” to pay tribute to all the body donors as well as use this opportunity to teach students to respect and appreciate their life (Fig. 1). This marks the beginning of humanistic values inculcation among our medical students. To date, more than 4000 medical students and teachers have participated in this ceremony. It has become a preserved activity on our anatomical calendar of events. Even several years after the event, many students have reported vivid memories along with their anticipation to see the cadaver and conquer the associated fears. The students listen to speeches about the lives of body donors, how bodies are donated and cared for as well as why they should respect them during their study. They are also informed about various voluntary positions in various committees such as the First Aid society, Museum Ushering, Qingming Tomb Sweeping Festival working group, and the body donation office. After the attendance at the “Square of Perfect Goodness”, the students then go through an orientation in the dissection rooms where they watch videos of the lives of now “silent mentors” while they were alive along with previous ceremonies. They are allowed to ask any questions that they may have at this time.

### The body donation office

A body donation office is affiliated to the Department of Human Anatomy of our School of Medicine. A special administrator in this body bequeathal program is responsible for introducing, registering, and accepting body donations. This administrator’s main responsibilities also include explaining the body donation procedures and partaking in ensuing logistical issues like admitting, registering, and providing quick instructions as well as helping those people willing to donate their bodies. To honor the body donors and to provide the donor’s family with an opportunity to commemorate their loved family members, our faculty organizes a memorial ceremony at a public cemetery. This ceremony, also attended by medical students, is conducted during every Chinese Qingming Festival. The Qingming Festival, also known as Tomb-Sweeping Day in English, is a traditional Chinese festival that falls on the first day of the fifth solar term of the traditional Chinese lunisolar calendar. Chinese families visit the tombs of their departed family members to clean the gravesites, pray to their ancestors, and make ritual offerings. On this day, the medical students, families of “silent mentors” and faculty join hands at the final resting place of the remains of the “silent mentors”. This practical opportunity is used to encourage the students and anatomy teachers to spread the idea of body donation and further promote public awareness of our body bequeathal program. The ceremony also creates a great deal of reflection on the part of the medical students, some of which would be important in ethical, humanistic, and professional practice.

The second part of the study focused on undergraduate medical students taking the Anatomy Course at Zhongshan School of Medicine, Sun Yat-sen University. The medical school runs a five-year undergraduate program under which the first (systemic anatomy) and third-year (regional anatomy) students take a total 180-h course in anatomy (traditional lectures and cadaver dissection). The ceremony to honor body donors is conducted at the beginning of the third year.

The five-point Likert-like scale questionnaire, adapted from da Rocha et al. [16] was used to evaluate the impact of a ceremony in honor of the body donors on ethical and humanistic attitudes of medical students (Supplementary Figure 1). It comprised of 36 questions allocated to three sections, covering demographic information of the medical students, cadaver dissection, and knowledge and reflections on the body bequeathal program and the ceremony in honor of body donors. It was delivered online to consenting third-year medical students (*n* = 500) and 171 returned filled within a 2 weeks deadline resulting in a response rate of 34.2%. All the participants who answered the questionnaire had to sign an informed consent form first. The researchers ensured anonymity and confidentiality both during the collection and in the analysis of the participants’ responses as the students were not required to enter any identifying information and this aspect was emphasized in the introduction of the study and questionnaire. The questionnaire was delivered online and there was no attempt to collect information on who responded to the questionnaire. Expert panel review was used to test and validate the questionnaire while its internal consistency was measured using Cronbach’s alpha.

### Statistical analysis

Data analysis was performed using Statistical Package for the Social Sciences Statistics v22 (IBM Inc., USA). The results were presented as means and standard deviation (±SD), frequency, and percentages. Associations and homogeneity were tested by the Chi-Squared test, Cochran’s Q-Test, or Fisher’s Exact Test, as indicated. Statistical significance was noted if *P* < 0.05. The differences between the groups (male-female, attendees-non-attenders) were tested using the two-tailed unpaired samples t-tests.

## Results

### Lessons from our Anatomico-humanist cultivation program

According to the local professors, an important aspect arising from the humanist-oriented curriculum system is the cultivation of medical humanistic qualities among learners as they study anatomy. A general misconception has always been that the cultivation of medical humanistic qualities is a prerogative of the courses such as introduction to medical practice, medical ethics, medical communication, and medical psychology. While philosophy, among other humanities areas, does promote medical humanistic qualities, the local professors believe that the deliberate approach taken during our anatomy classes plays a crucial role in cultivating the medical humanistic spirit and qualities.

### Focus on education quality and anatomists as mentors

According to local professors, anatomy instructors can play a crucial role in helping and influencing students through their words and deeds. Revelations from the in-depth interviews show that every new instructor in the anatomy department has to dissect a cadaver under the instruction of a senior professor. This chain of mentorship is extended to medical students taking anatomy classes as a core component of their pre-clinical medical training. Furthermore, cadaver dissection demonstrations conducted in the department have been geared to help the students understand the significance of dissection in their future performance in clinical and surgical procedures. The professors unanimously believed that the dissection laboratory is an ideal place to develop rigorous, responsible, and philanthropic attitudes of future doctors. It is in these laboratories that the instructors integrate medical humanistic knowledge into anatomy teaching content. For example, when introducing related knowledge, the medical students learn that some anatomists were once persecuted by the religious fraternity for insisting on their scientific theory that dissection is a gold standard in Medicine. The professors also noted that over the years the students have posed questions about the source of cadavers as well as their own physical and psychological well-being after cadaver exposure. Such reports have generated the need for the second part of this study which focused on how students view dissection after participation in interventions such as ceremonies to honor body donors. The anatomy professors also believed that accomplishing guided dissection with well-trained mentors nourishes the students’ passion for their ethical professional duties.

### Evaluation of the body donation program and ceremonies in honor of the body donors: class of 2017

One hundred seventy-one questionnaires were answered and returned by third-year medical students of which 72 (42.11%) were males and 99 (57.89%) were females. Their mean age was 20.6 (± 1.0). The homogeneity tests in all variables in different categories revealed no statistically significant differences, thus permitting them to be grouped together. The students’ participation in the ceremony ceremonies to honor body donors is not compulsory. Of the 171 respondents, 124 (72.51%) had participated in the body donation ceremony. Most of the students identified as atheists (150/171; 87.72%) and others were Buddhists/Taoists (10/171; 5.85%), Christians (3/171; 1.75%), Muslims (2/171), and Hindu (1/171).

### Dissection, death and dying, ethics and humanistic practice

**Fig. 2:**
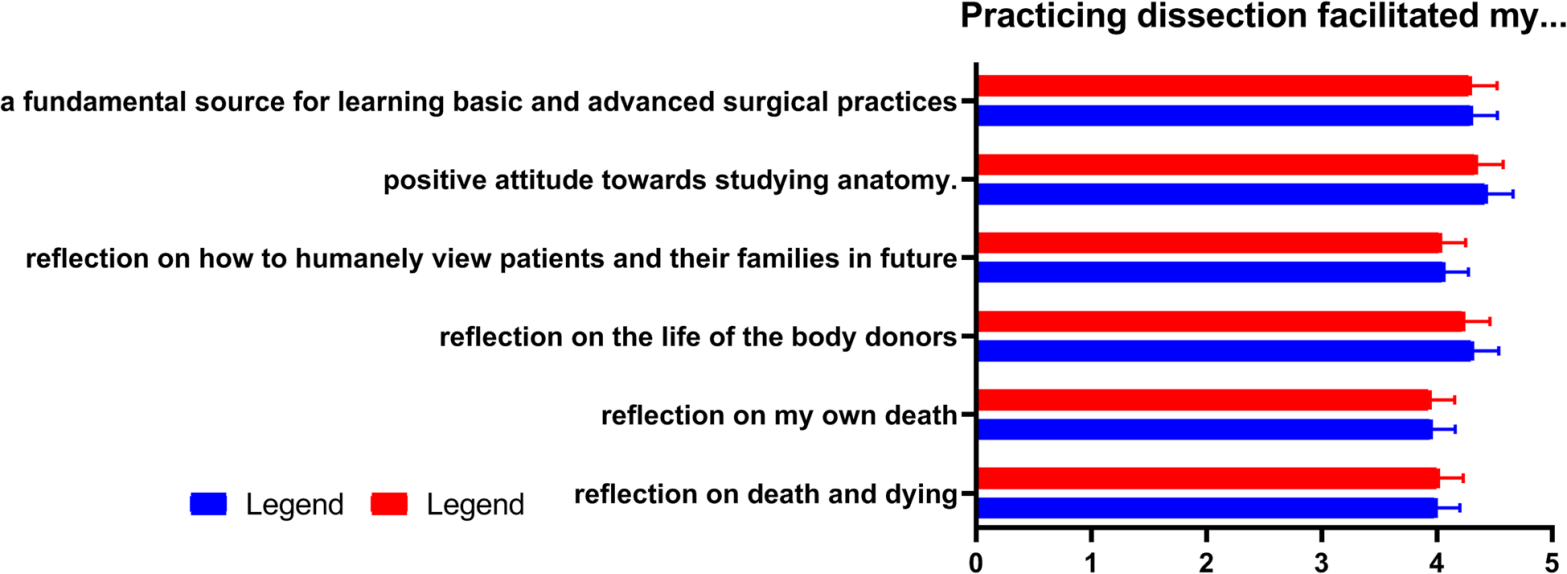
The results of students’ survey on the role of dissection on reflections of life, death, body donors, and studying anatomy. The graph shows the responses of all respondents divided into those who attended the ceremony (*n* = 124) and those who did not (*n* = 47)

The majority of students (116/171; 67.84%) had never seen a dead human body or cadaver before the start of medical training. However, most of them (161/171) knew that they would be dissecting cadavers as part of their medical training before enrolment. Most (80.75%) of the medical students surveyed reported that dissecting cadavers facilitated their reflection on death and dying while slightly less (77.20%) felt that it caused the reflection of their own death. These reflections did not discriminate against sex or whether or not a student had attended a ceremony to honor body donors (*P* > 0.05). Figure 2 below shows that, generally, medical students who had attended a ceremony to honor body donors marginally felt positively influenced by cadaver dissection. This positive influence was felt in terms of the attitude towards studying anatomy, how to humanely view future patients, as well as reflections on death and the lives of donors. In addition, 88.88% regard cadavers as a fundamental source for learning basic and advanced surgical practices.

The majority of the students (60.82%) strongly think that anatomical dissection of corpses in their anatomy course was important. Being aware that some people donate their bodies for use in teaching has increased the commitment to studying anatomy in 95.33% of them. Also, those students who attended the ceremony to honor body donors (4.56 ± 0.56, *n* = 124) were better influenced (committed to studying anatomy) by being aware that some people donate their bodies for use in medical school than those who elected not to attend the ceremonies (4.32 ± 0.76, *n* = 47). The difference was statistically significant (*P* = 0.022).

**Fig. 3:**
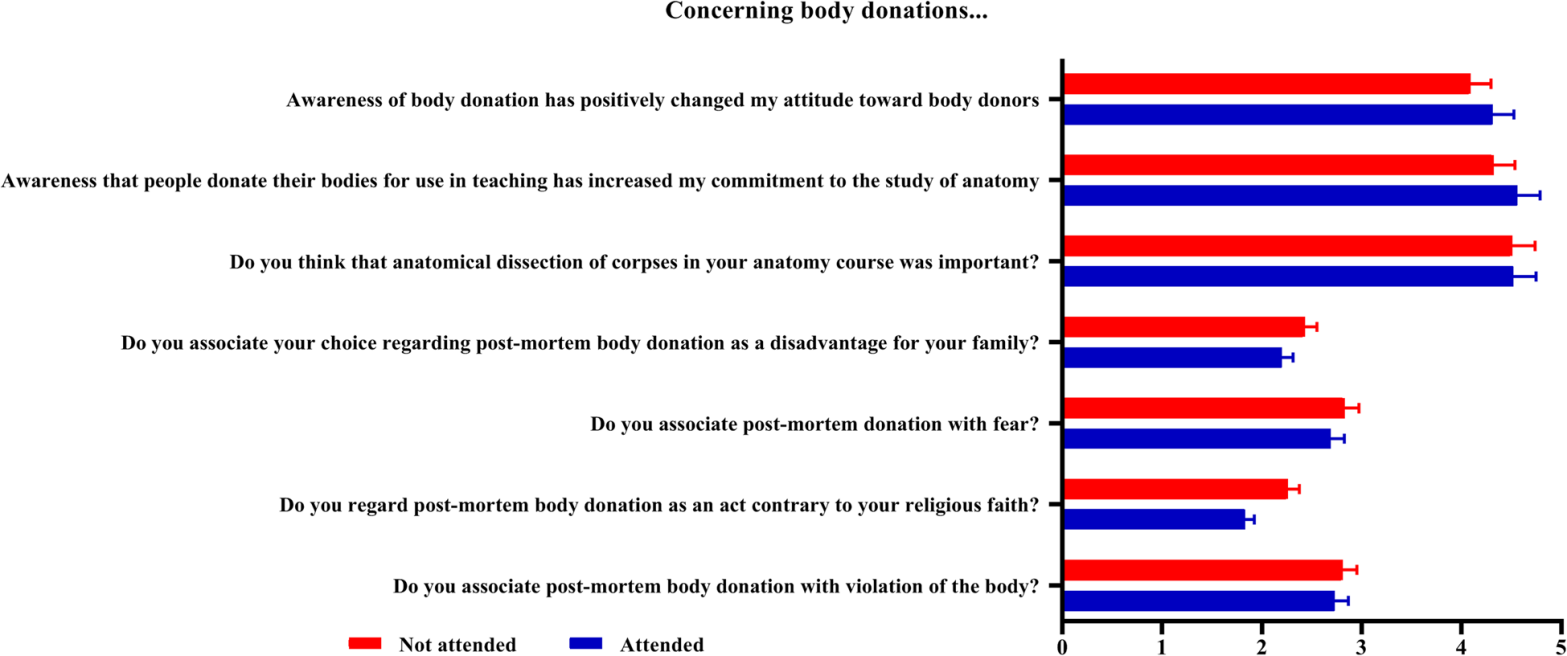
The perspectives of students towards the body donors and different ethical and humanistic attitudes. Mean calculated using Likert scale: 1 = strongly disagree, 2 = disagree, 3 = indifferent, 4 = agree, 5 = strongly agree

Nearly half the students did not associate post-mortem body donation with violation of the body (45.61% versus 31.58%), as an act contrary to their religious faith (75.44% versus 7.01%) or fear (46.2% versus 29.82%). However, the students who did not attend the gratitude ceremony for body donors generally felt that body donation acts were contrary to religion and violated the body. Therefore, they were afraid of it compared to their counterparts who attended the ceremony. However, the level of fear was significantly higher among those medical students who did not attend the ceremony (2.26 ± 1.05; *n* = 47) compared to those who attended (1.83 ± 0.91; *n* = 124), *P* = 0.010 (Fig. 3).

**Fig. 4:**
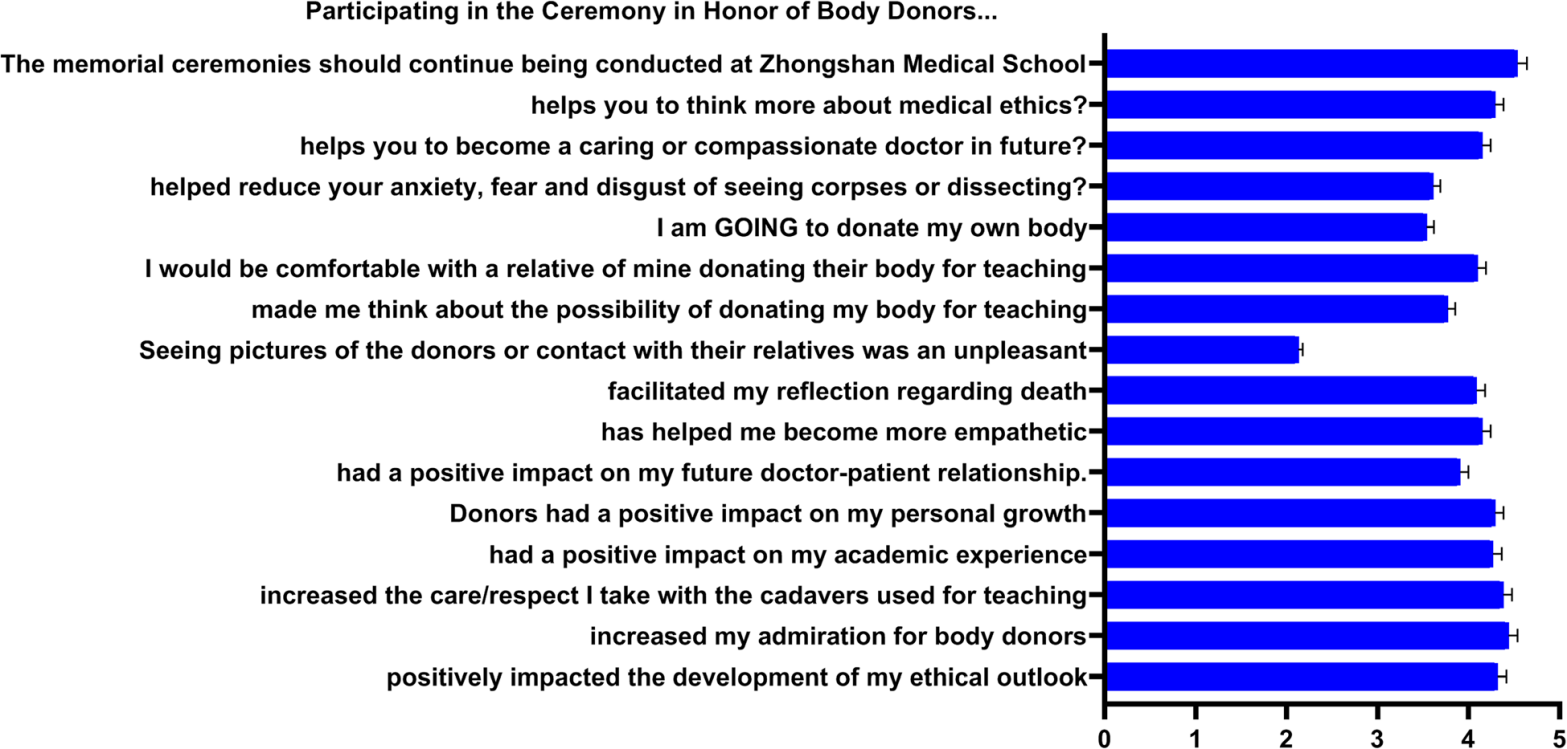
The perspectives of students towards the body donors and different ethical and humanistic attitudes after attending a ceremony to thank body donors. Mean calculated using Likert scale: 1 = strongly disagree, 2 = disagree, 3 = indifferent, 4 = agree, 5 = strongly agree

The perception of the medical students that attended the ceremony to honor the body donors with respect to the ethical and humanistic aspects was assessed as part of the wider evaluation of our University’s body bequeathal project evaluation (Fig. 4). About 72.51% (124/171) elected to attend the year’s gratitude ceremony for body donors. Most of these students strongly felt that attending the body donor gratitude ceremony greatly enhanced their admiration of donors, care to cadavers, positive impact on the academic experience, and personal growth. Personally, such an undertaking was reported to positively influence the students’ humanistic outlook in terms of medical ethics, being compassionate, empathetic, and having a better future doctor-patient relationship. Interestingly, the majority of the students felt that attending the ceremony caused them to reflect on matters such as death, dying and helped reduce anxiety, stress, as well as disgust associated with dissecting. Furthermore, the majority reported that pictures of donors or their relatives were not pleasant (90/124, 72.58%). Also, most felt they would be comfortable if their own relatives decided to donate their bodies to science (105/124; 84.68%). Slightly above half were affirmative that they would donate their bodies (54%) while nearly all of them (120/124, 96.77%) strongly felt that the ceremony conducted by our medical schools should continue being held.

## Discussion

Competencies within the medical practice such as knowledge and understanding of the basic, clinical, humanities, and relevant medical ethics are now being rethought and reflected upon to improve them [17]. Amongst all these crucial issues, we chose to address humanistic values education, a matter largely given little space in various medical anatomy curricula [18].

Recently, numerous studies have looked at the influence that the ceremonies to honor body donors run by body bequeathal programs have on medical students ([11, 14]; da Rocha et al., 2019). The majority of the ceremonies are held at the end of anatomy courses [19] while those held at the beginning [20], like ours, have preemptive benefits. Their general benefits were that students experienced improved attitudes towards death, dying, relations with patients, a sense of professional responsibility, and alleviation of negative emotions associated with dissections [20–22]. In this study, we sought to contribute to this body of knowledge by describing the gratitude rituals at Sun Yat-sen University’s medical school and also add students’ feedback further data on anatomists’ contributions to the ethical and humanistic aspects of medical training**.**

Anatomy, often divided into systemic anatomy and regional anatomy is taught early alongside other basic medical or preclinical sciences to medical students in most universities of China. This feat place anatomy at an optimum vantage point for introducing humanist and ethical principles to new students seeking to pursue careers in various medical specialties. The gross anatomy component that invariably involves cadavers is also ideal for introducing death and dying issues as well as alluding to humanistic and ethical values. In the present study, most of the students reported a positive influence of dissections on humanistic and ethical aspects such as reflecting about own death, that of body donors as well as having a compassionate and empathetic outlook when dealing with patients in the future. Also, the students felt that these views were further enhanced by attending the ceremony to honor body donors. Therefore, the benefits of introducing the humanistic and ethical component to anatomy appeared to reap some benefits for our cohort. Similar studies have reported the benefits of anatomy in this regard [23–27]. This is in stark contrast with the previously promoted approaches such as the “detached concern” which was thought to promote medical students’ well-being while dealing with cadavers [2, 3]. An effective way of reinforcing respect and compassion in medical students is to hold a memorial ceremony of gratitude towards the “silent mentors” [28]. Most importantly, a memorial ceremony such as one conducted at our institution provides a positive, intellectually, and emotionally intense introduction to a course in human dissection. Additionally, since a ceremony is experiential in nature, it is more effective than didactic methods in communicating values of respect and compassion [19, 29] as well as managing physical and psychological stress [30]. Since medical students are coming into contact with cadavers (many of them for the first time) they tend to grapple with psychological and physical effects of the exposure. These psychological effects include fainting, nausea, depression, insomnia, and symptoms resembling post-traumatic stress disorder [31–33]. The majority of the students in the present study reported experiencing less stress, anxiety, and disgust after attending a ceremony to honor donors. It appears such a preparation helped them to prepare for cadaver exposure and lessened the psychological burden. It is very important to pay attention to these even though they affect a small proportion and often die away with repeated cadaver exposure [8, 20, 32, 34, 35].

The local introduction of the concept of “silent mentor” started from a cooperation with the Tzu Chi University of Taiwan in 2008. As a forerunner to the ongoing humanistic approach to teaching, Tzu Chi University’s curriculum emphasizes empathy and respect for the body donors. Tzu Chi University students honor donors as “Silent mentors” [28, 36]. These “silent mentors” act as special teachers that help guide the students as they explore the human body. As aptly put by one professor, “beyond anatomy knowledge required for surgery, the silent mentors teach the medical students respect, gratitude, and compassion, values we have actively pursued in our anatomy courses”. Beginning in 2008, the cooperation with the medical school of Tzu Chi University has seen local professional faculty guiding many local medical students in a virtual surgery curriculum. Many students have since participated in similar programs and the professors reported that the students have been impressed by the humanistic spirit transmitted through the curriculum. The professors also believed these powerful experiences laid very strong foundations of their humanistic spirit as well as ethical qualities in our graduates. Therefore, the concepts are worth exporting elsewhere although they have never been formally tested.

The use of cadavers in the modern medical curriculum is under threat from those who believe that cadavers are potentially unsafe, disgusting, require skilled teachers, and require dissection rooms that are expensive to maintain at required laboratory safety standards [37, 38]. On the other hand, several others believe that their use may have benefits beyond just teaching, including teaching about medical fallibility and uncertainty, as well as raising issues of death and dying [39–41]. As recently reviewed by Ghosh [42], the dissection room, along with the rules and steps for receiving, use, and disposal of cadaver materials gives crucial room to nurture the ethical practice principles among medical students early in the curriculum. Our present study adds evidence in support of the utility of cadavers beyond the learning of anatomical facts but building humanist doctors. Using cadavers has long been touted as useful in instilling humanistic attitudes towards patient care [43].

In this study, medical students reported that exposure to the gratitude ceremony made them appreciate the sacrifices of body donors and also positively motivated them to have a good doctor-patient relationship in the future. The majority of the students (54%) felt motivated to donate their bodies after participating in the body donor appreciation ceremony. This finding is concordant with previous studies reporting that ranges from as high as 60% (da Rocha et al., 2019), 27% (Williams et al. [44], and a low of 7% (Alt-Epping et al. [45]. Within the general public of China, around 27.5% of respondents expressed a clear willingness to donate their bodies with the elderly less likely to agree thereby warranting re-assessing and re-interpreting Confucianism beliefs regarding body donation [13]. As noted by da Rocha et al., (2019), the present data suggested that the donor appreciation ceremonies represent a potential opportunity to cultivate feelings of altruism related to the act of body donation among medical students. Apart from the use of dissection for students to grasp a clear visuospatial picture of the organization of the human body, it also helps with experiencing the texture of human tissues, fostering teamwork, promoting professional development, and also helps them come to terms with the death and dying [1]. In the present study, feedback from students revealed that participation in ceremonies enhanced their commitment to studying, appreciating the value of dissection, personal growth, and strengthening of compassion. All these benefits of engagement in ceremonies have been reported in a variety of settings elsewhere [35] thereby placing anatomy at the core of early inculcation of humanist values in preclinical medical training [42]. Although most studies reported lacking a formal curriculum [27, 46], the utility of such programs continues to appear frequently in recent literature [47]. Herein, we reflected on our practical steps towards fostering humanistic medical practice using gross anatomy dissections and gratitude ceremony rituals early in the anatomy curriculum. As we narrated our steps towards building humanist conscious doctors, we highlighted the crucial elements from the students’ feedback that could be utilized when teaching, evaluating, and promoting humanist values available in the cadaver dissection laboratory.

The local professors noted many ways designed to make learning extend outside the classroom in order to cultivate the humanistic values in the anatomical courses of the local School of Medicine. One of them was the setting up of a students’ Body Donation Society whose mandate is to expound the idea of humanity, love, and dedication to the improvement of medical training to the general population. The medical students in this society introduce the significance and procedures of body donation to the public during school holidays. During these times, they spread the messages of love and dedication of the “Silent Mentors” and make the public understand the objective and significance of the body donation to medical training and healthcare provision in general.

In addition, a first aid promotion team, which is affiliated to the department of anatomy, was started by the First Aid Volunteer Society. Students in this society help to explain and publicize relevant first aid knowledge to the public at the Science Museum or in different communities around our University’s city. Furthermore, we also have some medical students in the Outside-Classroom Science Society who also visit the nearby communities to publicize correct health ideals, body donation programs, and popularize basic medical knowledge. The anatomy department also hosts a modern medical museum that integrates multiple-subjects, high-end technology, networking, and resource sharing. The medical museum is home to a wide range of cadaveric specimens for all university programs to study. It is open to the general public receiving over 6000 visits per year, thus, popularizing medical science education as well as body donation in our city and province. The volunteer medical students work as ushers and tour guides at the medical museum. They get to enhance their sense of social responsibility as well as opportunities to apply their humanistic values in real-life situations well before they start patient engagement.

### Limitations of the study

The response rate was generally low (34.2%) probably due to the relatively shorter time frame during which they were supposed to return completed questionnaires. This may also have led to a lesser number of medical students (27.49%) who had not participated in the gratitude ceremony to honor body donors. In addition, this study was based on a single-center and was a cross-sectional survey directed as a single class. Future studies should consider multi-center surveys that involve other data gathering methods such as in-depth interviews, focused group discussions, and longitudinal follow-ups. This would enable establish if there is any long-term impact of introducing humanistic and ethical principles in human anatomy teaching early on in the curriculum.

## Conclusion

A load of cultivating medical humanistic qualities is heavy and could be shared among different units within the medical schools. Imperceptible influence through the combination of the basic medical curriculum and outside-classroom activities might help to accomplish the goal of cultivating high-quality medical students and professionals for the future. This positive feedback about the practical approach to promote humanism from our school of medicine provides novel insights that could help other interventions aimed at promoting humanistic competencies in the anatomy laboratory experience. The long-term benefits would be a medical graduate who exudes empathy, relates well with patients and their significant others leading to a productive doctor-patient relationship.

## Supplementary Information

**Additional file 1.**

## Data Availability

The datasets used and/or analysed during the current study are available from the corresponding authors on reasonable request.
