# Supplementary material for: Cultivation of humanistic values in medical education through anatomy pedagogy and gratitude ceremony for body donors
Source: BMC Med Educ. 2020 Nov 17;20:440. doi: 10.1186/s12909-020-02292-1 (PMC7672936; doi:10.1186/s12909-020-02292-1)
Supplement: Supplementary file 1 — Additional file 1. [file 12909_2020_2292_MOESM1_ESM.docx]

| Demographics | **Please answer all the questions truthfully.**  **SEX:** ( ) Male ( ) Female  **AGE:________ YEARS**  **COURSE YEAR: _______**  **Which of the following best describes your religious beliefs?**  ( ) Atheist ( ) Buddhist/Taoist ( ) Christian ( ) Hindu ( ) Jewish ( ) Muslim/Islam ( ) Other__________ |
| --- | --- |
| Practice of Dissection | **Had you seen a human corpse or dead body before coming to start medical training?**  ( ) Yes ( ) No  **Did you know that you will be dissection real human bodies before coming to Zhongshan Medical School?**  ( ) Yes ( ) No  **Practicing dissection facilitated my reflection on death and dying.**  ( ) strongly disagree ( ) disagree ( ) indifferent ( ) agree ( ) strongly agree  **Practicing dissection facilitated my reflection on my own death.**  ( ) strongly disagree ( ) disagree ( ) indifferent ( ) agree ( ) strongly agree  **Practicing dissection facilitated my reflection on the life of the body donors.**  ( ) strongly disagree ( ) disagree ( ) indifferent ( ) agree ( ) strongly agree  **Practicing dissection facilitated my reflection on how to humanely view patients and their families in future.**  ( ) strongly disagree ( ) disagree ( ) indifferent ( ) agree ( ) strongly agree  **Practicing dissection facilitated my positive attitude towards studying anatomy.**  ( ) strongly disagree ( ) disagree ( ) indifferent ( ) agree ( ) strongly agree  **Do you regard cadavers as a fundamental source for learning basic and advanced surgical practices?**  ( ) strongly disagree ( ) disagree ( ) indifferent ( ) agree ( ) strongly agree  **Do you associate post-mortem body donation with violation of the body?**  ( ) strongly disagree ( ) disagree ( ) indifferent ( ) agree ( ) strongly agree  **Do you regard post-mortem body donation as an act contrary to your religious faith?**  ( ) strongly disagree ( ) disagree ( ) indifferent ( ) agree ( ) strongly agree  **Do you associate post-mortem donation with fear?**  ( ) strongly disagree ( ) disagree ( ) indifferent ( ) agree ( ) strongly agree  **Do you associate your choice regarding post-mortem body donation as a disadvantage for your family?**  ( ) strongly disagree ( ) disagree ( ) indifferent ( ) agree ( ) strongly agree  **Do you think that anatomical dissection of corpses in your anatomy course was important?**  ( ) strongly disagree ( ) disagree ( ) indifferent ( ) agree ( ) strongly agree |
| Awareness of body donation | **Awareness that people donate their bodies for use in teaching has increased my commitment to the study of anatomy**.  ( ) strongly disagree ( ) disagree ( ) indifferent ( ) agree ( ) strongly agree  **Awareness of body donation has positively changed my attitude toward body donors.**  ( ) strongly disagree ( ) disagree ( ) indifferent ( ) agree ( ) strongly agree  **Gaining a better understanding of the donor’s selfless gesture positively impacted the development of my ethical outlook.**  ( ) strongly disagree ( ) disagree ( ) indifferent ( ) agree ( ) strongly agree |
| Ceremony in Honor of the Body Donors | **Have you participated in any edition of the Ceremony in Honor of Body Donors? (If you have never participated, Please stop here)**  ( ) Yes ( ) No  **Participating in the Ceremony in Honor of Body Donors increased my admiration for body donors.**  ( ) strongly disagree ( ) disagree ( ) indifferent ( ) agree ( ) strongly agree  **Participating in the** **Ceremony in Honor of Body Donors increased the care/respect I take with the cadavers used for teaching.**  ( ) strongly disagree ( ) disagree ( ) indifferent ( ) agree ( ) strongly agree  **The Ceremony in Honor of Body Donors had a positive impact on my academic experience.**  ( ) strongly disagree ( ) disagree ( ) indifferent ( ) agree ( ) strongly agree  **The Ceremony in Honor of Body Donors had a positive impact on my personal growth.**  ( ) strongly disagree ( ) disagree ( ) indifferent ( ) agree ( ) strongly agree  **The Ceremony in Honor of Body Donors had a positive impact on my future doctor-patient relationship.**  ( ) strongly disagree ( ) disagree ( ) indifferent ( ) agree ( ) strongly agree  **Taking part in the** Ceremony **in Honor of Body Donors has helped me become more empathetic.**  ( ) strongly disagree ( ) disagree ( ) indifferent ( ) agree ( ) strongly agree  **The Ceremony in Honor of Body Donors facilitated my reflection regarding death.**  ( ) strongly disagree ( ) disagree ( ) indifferent ( ) agree ( ) strongly agree  **Seeing pictures of the donors and having contact with their relatives during the** Ceremony **in Honor of Body Donors was an unpleasant experience.**  ( ) strongly disagree ( ) disagree ( ) indifferent ( ) agree ( ) strongly agree  **Attending the Ceremony in Honor of Body Donors made me think about the possibility of donating my body for teaching.**  ( ) strongly disagree ( ) disagree ( ) indifferent ( ) agree ( ) strongly agree.  **After attending the Ceremony in Honor of Body Donors I would be comfortable with a relative of mine donating their body for teaching.**  ( ) strongly disagree ( ) disagree ( ) indifferent ( ) agree ( ) strongly agree  **After attending the Ceremony in Honor of Body Donors I am GOING to donate my own body**  ( ) strongly disagree ( ) disagree ( ) indifferent ( ) agree ( ) strongly agree  **Do you think attending the Ceremony in Honor of Body Donors helped reduce your anxiety, fear and disgust of seeing corpses or dissecting?**  ( ) strongly disagree ( ) disagree ( ) indifferent ( ) agree ( ) strongly agree  **Attending the Ceremony in Honor of Body Donors helps you to become a caring or compassionate doctor in future?**  ( ) strongly disagree ( ) disagree ( ) indifferent ( ) agree ( ) strongly agree  **Attending the Ceremony in Honor of Body Donors helps you to think more about medical ethics?**  ( ) strongly disagree ( ) disagree ( ) indifferent ( ) agree ( ) strongly agree  **The memorial ceremonies should continue being conducted at Zhongshan Medical School**  ( ) strongly disagree ( ) disagree ( ) indifferent ( ) agree ( ) strongly agree  **How did these ceremonies be improved?**  **______________________________________________________________________________________________________________________________________________________________________________________________________________________________________________________________________________________________________________________**  **The end: Thank you for Participating!** |
